# Midwifery students’ attitudes toward violence against women by metaphor analysis: a qualitative study

**DOI:** 10.1590/1806-9282.20260494

**Published:** 2026-08-28

**Authors:** Selma Sen, Sezer Er Güneri, Huriye Akkale

**Affiliations:** 1 Manisa Celal Bayar University, Faculty of Health Sciences, Department of Midwifery – Manisa, Turkey.; 2 Ege University, Faculty of Nursing, Department of Women’s Health and Diseases Nursing – İzmir, Turkey.; 3 Manisa Celal Bayar University, Faculty of Sciences, Department of Midwifery – Manisa, Turkey.

**Keywords:** Gender-based violence, Students, Attitudes, Metaphors.

## Abstract

**OBJECTIVE:**

We conducted this study to examine the attitudes and metaphorical perceptions of violence against women among midwifery students.

**METHODS:**

This Convergent Mixed-Methods study involved 383 midwifery students enrolled in a health sciences department during the 2024–2025 academic year. Data were collected using the Student Information Form, the Scale of Attitude Toward Violence Against Women, and the Metaphor Identification Form on Violence Against Women.

**RESULTS:**

Of the midwifery students, 21.5% reported exposure to at least one form of violence, with emotional and verbal violence being the most commonly reported. A substantial proportion of midwifery students remained silent when exposed to violence. The total mean attitude score for violence against women was 27.32±6.46, indicating non-traditional and non-myth-supporting attitudes. The metaphor analysis yielded eight overarching themes.

**CONCLUSION:**

The findings suggest that midwifery students hold contemporary and critical attitudes toward violence against women; however, their help-seeking behaviors remain limited due to sociocultural barriers. The metaphors produced reflect a deep understanding of the destructive, cyclical, and structurally oppressive nature of violence. Overall, our findings demonstrate that midwifery students possess a multidimensional and detailed understanding of violence against women; however, persistent barriers to help-seeking behavior indicate that sociocultural norms remain powerful.

## INTRODUCTION

Violence against women (VAW) is internationally recognized as one of the most pervasive violations of human rights and is a ­critical public health issue. The World Health Organization^1^ reported that nearly one in three women worldwide experiences physical and/or sexual violence during their lifetime, most commonly perpetrated by an intimate partner. Such violence has profound consequences for women’s physical, psychological, and social well-being and is deeply rooted in gendered power inequalities^2^.

Methodologically, systematic metaphor identification procedures, particularly the Metaphor Identification Procedure ­developed by the Pragglejaz Group (2007) and its later adaptation, the Metaphor Identification Procedure Vrije Universiteit^3,4^, have strengthened the reliability and validity of metaphor research.

Beyond its epidemiological dimensions, scholars are ­increasingly arguing that how people conceptualize and talk about violence significantly shapes their social attitudes, normative expectations, and responses to abuse. Language is not a neutral medium; rather, it is used in the construction of and reflects underlying belief systems. In this regard, metaphors play a central role. According to conceptual metaphor theory (CMT), metaphors are cognitive structures that allow individuals to understand an abstract or complex phenomenon in terms of a more familiar phenomenon^5^. Therefore, metaphors can illuminate, obscure, justify, or challenge social problems, including VAW.

Exploring students’ metaphorical perceptions of VAW is especially valuable because young adults both reflect and shape contemporary sociocultural norms. Identifying these ­metaphorical patterns can thus help educators and ­policymakers design more effective prevention strategies, awareness programs, and curricular initiatives.

Metaphor analysis offers a powerful lens through which to understand how students cognitively frame VAW. By ­examining these metaphorical constructions, we aim to contribute to a deeper understanding of young adults’ perceptions of and the broader discourse surrounding gender-based violence. Specifically, we examine the attitudes and metaphorical perceptions of VAW among midwifery students who will enter the midwifery profession in the near future.

## METHODS

### Study design and aim

This Convergent Mixed-Methods study was conducted to examine the attitudes toward and metaphorical perceptions of VAW among midwifery students who will enter the midwifery profession in the near future.

### Study population and sample

The study population comprised all students enrolled in the Midwifery Department of the Faculty of Health Sciences at the university (N=411). No sampling method was employed since we aimed to reach the entire population. After reaching 93% of the population (n=383), data collection was concluded.

### Data collection tools

#### Student information form

The Student Information Form was developed to assess the sociodemographic characteristics of midwifery students and their parents. The form also includes 25 items assessing midwifery students’ exposure to or experiences with violence.

#### Scale of attitude toward violence against women

Attitudes toward VAW were measured using the Scale of Attitude Toward Violence Against Women, developed by Gombul (2000). The scale comprises 19 Likert-type items. The scale uses a five-point Likert format, with responses ranging from 1=strongly disagree to 5=strongly agree.

In our study, the Cronbach’s alpha coefficient was found to be 0.85. The scale includes four subdimensions: Economic violence, Emotional, psychological, and sexual violence, Legitimizing myths, and Rationalizing myths. Six items (7, 8, 10–13) are reverse-coded. The total possible score ranges from 19 to 95. High scores signify an increase in negative attitude toward violence, and low scores signify a positive attitude. The scale has no cut-off point^6^.

#### Metaphor identification form for VAW

The Metaphor Identification Form was developed by ­examining national and international literature on metaphor analysis^7^. The form comprises two open-ended prompts designed to elicit midwifery students’ metaphorical perceptions of violence.

### Data collection procedure

Before their application, two experts holding doctoral degrees in their respective fields reviewed all data collection tools to ensure content validity. A pilot study was then conducted with five midwifery students to test clarity and comprehensibility. Necessary revisions were made based on their feedback, and the forms were finalized.

Midwifery students were informed about the aims and procedures of the research in face-to-face classroom sessions during scheduled course hours. The forms, including the metaphor identification component, were distributed after detailed instructions were provided. Completing the forms took ­approximately 15 min.

### Data analysis

#### Quantitative analysis

Quantitative data were analyzed using SPSS version 24.0. Descriptive statistics, including frequencies, percentages, means, and minimum and maximum values, were calculated.

#### Metaphor analysis model

The metaphors for VAW that students produced were analyzed using CMT, which conceptualizes metaphors as cognitive structures that help individuals understand abstract phenomena through more concrete and familiar experiences. Within this framework, VAW, an abstract and multifaceted social problem, is interpreted through the concrete source domains that students select^8^. As a result of this combined analytical framework, metaphors were categorized into thematic structures representing students’ perceptions of VAW. These themes captured how students conceptualized violence in terms of destruction, oppression, psychological deterioration, restriction, social invisibility, and other related dimensions. The process ensured analytical rigor since we validated themes through frequent comparisons, references to the literature, and internal consistency checks.

To ensure analytical rigor, two independent researchers coded the data separately. The inter-coder agreement rate was 90%, and the Cohen’s Kappa value was κ=0.84, indicating a high level of agreement between coders. Discrepancies in coding were discussed and resolved through consensus.

## RESULTS

It was determined that 47.0% of participants were in the 21–24 year age group, with a mean age of 21.00±1.92. Of our participants, 21.5% reported having been subjected to violence, 26.8% to emotional violence, and 19.5% to verbal violence. When asked what they did following the act of violence, 37.8% said they remained silent (Table 1).

**Table 1:** Students’ violence experience status.

	Yes		No	
	n	%	n	%
*Exposure to domestic violence	82	21.5	300	78.5
Types of violence**				
Physical violence	1	1.2		
Emotional violence	22	26.8		
Verbal violence	16	19.5		
Sexual violence	1	1.2		
Emotional, verbal violence	15	18.2		
Emotional, physical, verbal, economic violence	27	32.9		
Behavior being displayed when exposed to violence*				
I kept silent	31	37.8		
She/he said she/he was sorry and I was reconciled	24	29.2		
I went to the police station	1	1.2		
I left home	2	2.4		
Other	33	40.2		

*The data that were acquired in the table show the verbal statements of individuals, and no scale was used.

**Among the types of violence being committed, more than one option was marked. Percentages calculated based on n=82 students who reported exposure to violence.

When participants’ general attitudes toward VAW and their subgroup mean scores were examined, we found that the total mean score was 27.32±6.46; the mean score of Attitude Toward Economic Violence was 9.35±2.92; the mean score of Attitude Toward Emotional, Psychological, and Sexual Violence was 9.79±4.21; the mean score of Attitude Toward Legitimizing Myths was 3.33±0.96; the mean score of Attitude Toward Rationalizing Myths was 4.99±1.85 (Table 2).

**Table 2:** Students’ general attitudes toward violence against women and subgroup average scores.

Subgroups	Number of items	Min-max	X±SD
Attitude toward economic violence	7	8-33	9.35±2.92
Attitude toward emotional, psychological, sexual violence	6	7-26	9.79±4.21
Attitude toward legitimizing myths	3	5-15	3.33±0.96
Attitude toward rationalizing myths	3	4-29	4.99±1.85
Total scale score	19	40-76	27.32±6.46

SD: standard deviation.

That is, participants’ attitudes toward VAW moved away from the traditional view that violence perpetrated by men against women is normal behavior and moved closer to the contemporary view that supports women who are subjected to violence.

**Figure 1:**
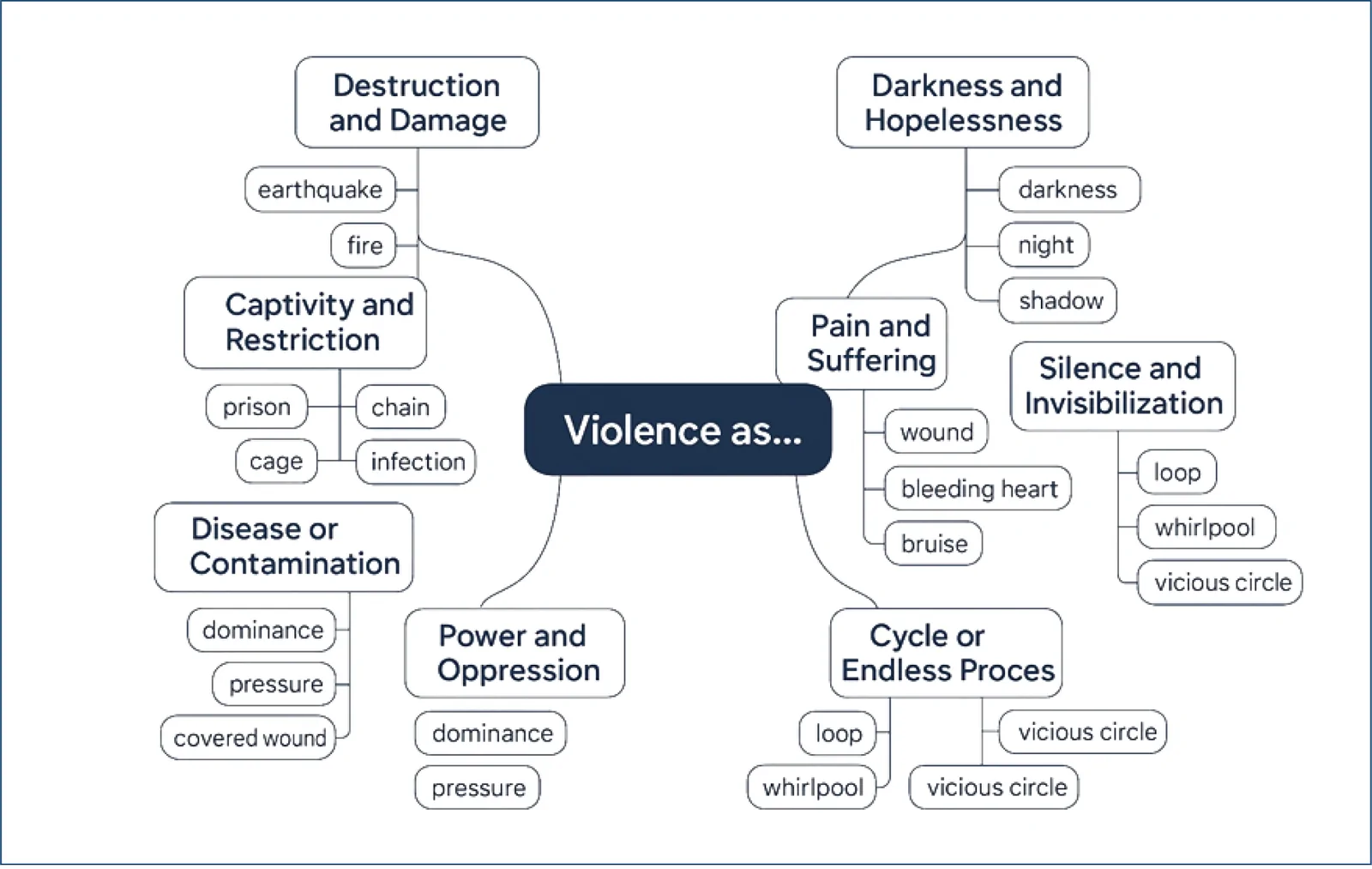
Metaphorical themes concept map.

A total of 383 midwifery students produced metaphors describing their perceptions of VAW. Following the conceptual metaphor analysis procedure, eight main themes were generated based on the semantic commonalities among the metaphors and their accompanying explanations (Figure 1).

### Theme 1: Violence as destruction and damage

Many participants conceptualized VAW as a force that breaks, crushes, or destroys. Metaphors such as “earthquake,” “fire,” “collapse,” “destruction,” and “broken glass” were mentioned, emphasizing the irreversible physical and psychological harm caused by violence. These metaphors also reflected the perception that the effects of violence are long-lasting and difficult to repair.

### Theme 2: Violence as darkness and hopelessness

Participants frequently used metaphors depicting violence as “darkness,” “night,” “a shadow,” or “a black hole.” These metaphors emphasized the sense of fear, isolation, and emotional deterioration associated with violence. The image of “darkness” symbolized both the hidden, unspoken nature of domestic violence and the psychological entrapment that prevents women from seeking help.

### Theme 3: Violence as captivity and restriction

Theme 3 included metaphors such as “prison,” “chain,” “cage,” and “handcuffs,” which conveyed the idea that violence limits women’s autonomy and freedom. Participants emphasized that victims often feel trapped, controlled, or unable to escape due to social pressure, economic dependency, or fear. This theme reflected the understanding that violence is not only physical but also structural and controlling.

### Theme 4: Violence as pain and suffering

Metaphors such as “wound,” “bleeding heart,” “bruise,” and “burning” appeared frequently. These metaphors foregrounded the embodied nature of violence, representing both physical pain and emotional suffering. Participants’ explanations revealed that even metaphors referring to physical injury were often meant to symbolize deeper psychological trauma, suggesting an integrated perception of bodily and emotional harm.

### Theme 5: Violence as ıllness or contamination

Some participants described violence using illness-related ­metaphors such as “cancer,” “virus,” “infection,” or “poison.” These metaphors portrayed violence as a phenomenon that spreads, worsens over time, and affects not only the ­individual but also their entire ­family and society. This theme aligned with public health perspectives on violence as a preventable yet pervasive social problem.

### Theme 6: Violence as power and oppression

Metaphors including “dominance,” “pressure,” “weight,” and “mountain on one’s shoulders” conveyed the perception of violence as a tool of patriarchal control. Participants emphasized gender inequality, cultural norms, and stereotypes as underlying mechanisms that sustain male dominance. This theme reflected the sociocultural dimension of violence.

### Theme 7: Violence as silence and invisibilization

Another group of metaphors, including “invisible wall,” “mute voice,” “covered wound” and “hidden storm,” reflected the hidden and normalized aspects of domestic violence. Participants emphasized societal denial, stigma, and the difficulty many women face in speaking about their experiences. Explanations often indicated that victims remain silent “to avoid judgment” or “due to fear of worsening the situation.”

### Theme 8: Violence as a cycle or endless process

Metaphors such as “loop,” “whirlpool,” “vicious circle,” and “spiral” depicted violence as repetitive and difficult to escape. Participants’ explanations revealed an awareness of the cyclical nature of domestic violence, including the escalation, reconciliation, and reoccurrence phases. This theme demonstrated that participants perceive violence not as an isolated event but as an ongoing pattern embedded within familial dynamics.

Overall, the metaphors that participants produced indicated that VAW can be conceptualized as a multifaceted problem involving destruction, psychological darkness, restriction of freedom, physical and emotional suffering, illness-like spread, power-based oppression, societal silencing, and cyclical continuation. The diversity of metaphors demonstrated that participants possessed a complex and multilayered understanding of violence, reflecting both individual experiences and sociocultural perspectives.

## DISCUSSION

Integrating quantitative and qualitative findings gave us a more comprehensive understanding of how future healthcare professionals interpret VAW.

Our quantitative results show that 21.5% of participants have experienced violence, with emotional and verbal violence being the most common types. This aligns with the findings of previous studies indicating that emotional and psychological violence is more frequently reported than physical violence among young adults^9,10^.

A concerning finding was that 37.8% of participants remained silent when exposed to violence, and only 1.2% sought help from legal authorities. This mirrors global evidence showing that many women, especially adolescents and young adults, hesitate to report violence due to fear, stigma, or familial pressure^10,11^.

The results for the Scale of Attitude toward Violence against Women demonstrate that participants generally hold non-traditional, non-myth-supporting views of VAW. These findings are consistent with those of studies showing that increased education, especially in health-related fields, is associated with more egalitarian and non-traditional attitudes toward gender-based violence^12,13,14^.

The metaphor analysis enriched our findings by revealing the cognitive and emotional frameworks guiding participants’ perceptions. The most prominent metaphors conceptualized violence as “destruction,” “darkness,” “captivity,” “pain,” “illness,” “oppression,” “silence,” and “a vicious cycle.” For instance, metaphors framing violence as “damage,” “wound,” or “fire” reflect the trauma and long-term psychological consequences of abuse^15^. The theme of violence as “a cycle” aligns with the concept of the cycle of violence. Meanwhile, metaphors describing violence as a “virus” or “epidemic” reflect the public health framing of gender-based violence as a preventable yet pervasive societal problem^10^. The theme of violence as power and oppression demonstrates participants’ understanding of gender inequality as a root cause of VAW, consistent with sociological and feminist frameworks emphasizing structural oppression over individual pathology in VAW^16^. Participants’ metaphors thus indicate a sophisticated grasp of the social determinants of violence.

A critical implication of the high rate of silence (37.8%) is the tension between emerging professional identity and personal vulnerability among midwifery students. Although midwifery students are trained to recognize and respond to violence in patients, they may experience difficulty applying the same principles to themselves. This paradox can be interpreted through the lens of professional socialization in healthcare, where expectations of resilience, emotional control, and competence may discourage help-seeking behaviors. Midwifery students may fear being perceived as weak, unfit for clinical responsibility, or professionally compromised if they disclose experiences of violence. In addition, concerns about stigma, confidentiality, and potential academic or clinical repercussions may further inhibit disclosure.

Overall, our findings demonstrate that at this university, midwifery students possess a multidimensional and detailed understanding of VAW; however, persistent barriers to help-seeking behavior indicate that sociocultural norms remain powerful.

## CONCLUSION

The quantitative findings demonstrated that although a significant proportion of midwifery students had been exposed to emotional or verbal forms of violence, their overall attitudes toward VAW were predominantly non-traditional and rejected societal myths that normalize male-perpetrated violence. These results suggest a shifting perspective among future midwives toward greater gender sensitivity and support for victims.

The qualitative metaphor analysis further revealed that midwifery students perceive VAW as a multidimensional and deeply harmful experience, characterized by destruction, darkness, captivity, pain, contamination, oppression, silence, and cyclical continuation. These metaphorical expressions reflect not only an intellectual understanding of VAW but also an emotional and moral recognition of its severity. The convergence between quantitative attitudes and qualitative metaphors underscores how midwifery students internalize violence as both a personal and societal issue.

Integrating reflective methods such as metaphor analysis – into training may further enhance midwifery students’ awareness of their own assumptions and emotional responses. Overall, this study contributes to the growing literature on healthcare midwifery students’ perceptions of VAW and offers important implications for midwifery education and practice.

## Data Availability

The datasets generated and/or analyzed during the current study are available from the corresponding author upon reasonable request.
